# Latent Tuberculosis Infection Treatment Completion while Shifting Prescription from Isoniazid-Only to Rifampicin-Containing Regimens: A Two-Decade Experience in Milan, Italy

**DOI:** 10.3390/jcm9010101

**Published:** 2019-12-31

**Authors:** Simone Villa, Maurizio Ferrarese, Giovanni Sotgiu, Paola Francesca Castellotti, Laura Saderi, Cecilia Grecchi, Matteo Saporiti, Mario Raviglione, Luigi Ruffo Codecasa

**Affiliations:** 1 StopTB Italia Onlus, 20159 Milan, Italy; 2 Regional TB Reference Centre, Villa Marelli Institute/ASST Niguarda, 20159 Milan, Italy; 3 Clinical Epidemiology and Medical Statistics Unit, Department of Medical, Surgical and Experimental Sciences, University of Sassari, 07100 Sassari, Italy; 4 Infectious Diseases Unit, IRCCS San Matteo, 27100 Pavia, Italy; 5 Department of Internal Medicine and Therapeutics, University of Pavia, 27100 Pavia, Italy; 6 Global Health Center, Multidisciplinary Research in Health Science University of Milan, 20122 Milan, Italy

**Keywords:** latent tuberculosis infection, preventive therapy, treatment completion, treatment dropout

## Abstract

To tackle the tuberculosis (TB) epidemic, in 2014 the World Health Organization launched the End TB Strategy, which includes action to prevent latent TB infection (LTBI) reactivation. Available preventive treatments (PT) are based on either isoniazid (INH) alone or rifampicin (RIF)-containing regimens. This study aims to assess and compare PT completion rates, the occurrence of adverse events, and the time of dropout among those receiving INH-alone or RIF-containing regimens at Villa Marelli Institute, Milan, Italy, covering the period from 1992 to 2018. A total of 19,670 subjects, belonging to various risk groups—mainly young (median age of 29 years), foreign-born (73.3%), and males (58.8%)—with presumed LTBI were prescribed PT (79.3% INH-alone and 20.7% RIF-containing regimens). The treatment completion rate was 79.4% on average, with higher rates among those receiving RIF-containing regimens (85.6%) compared to those that were prescribed INH-alone (77.8%) (*p* < 0.0001). Notably, some of the high-risk groups for progression of LTBI were more likely to complete PT from RIF-containing regimens. These groups included recent TB contact (89.9%, *p* < 0.0001), healthcare workers (93.5%, *p* < 0.0001), and homeless people (76.6%, *p* < 0.0001). Irrespectively of the chosen PT regimen, most of the dropouts occurred between the start of the treatment and the first follow-up visit (14.3%, 15.2% for those on INH-alone vs. 11.1% for those on RIF-containing regimens). Further shortening of the PT regimen is therefore an aim to ensure adherence, even though it might need further efforts to enhance the patient’s attitude towards starting and carrying out PT.

## 1. Introduction

Tuberculosis (TB) is a major global health issue, with 10.0 million (a range of 9.0–11.1) estimated incident cases per year, and is one of the top ten causes of death, accounting for ~1.5 million of deaths in 2018 [1]. 

The World Health Organisation (WHO) set targets in 2014 to end the global TB epidemic by 2030 with its End TB Strategy, which involves: reducing TB incidence by 80%, reducing mortality by 90%, and eliminating catastrophic costs for affected households. Thus, it is crucial to prevent the occurrence of disease by treating individuals with latent TB infection (LTBI) [2], especially those with the highest risk of developing TB (e.g., people living with human immunodeficiency virus (HIV)) [3]. Estimates counts that ~1.7 billion people were latently infected by *Mycobacterium tuberculosis* in 2014 [4]. 

In settings with a low annual TB incidence (<10 cases per 100,000 population) current WHO guidelines recommend preventive therapy (PT)—which can decrease TB occurrence by 60–90% [5]—for people living with HIV, for adult contacts of TB patients, and for other clinical risk groups (e.g., homeless persons) [6]. Furthermore, depending on the migratory flows, a country may implement screening programs and LTBI PT for those who had recently arrived (e.g., asylum seekers/refugees) [7,8]. 

The recommended standard treatment for LTBI is based on the administration of isoniazid (INH) for six or nine months [9]. However, a poor completion rate has been reported [10] for INH’s long duration and the occurrence of adverse events (AEs). Treatment based on rifamycins (e.g., rifampicin (RIF), rifapentine (RPT)) can shorten PT to 3–4 months [11,12]. Those regimens might cause less AEs and, consequently, improve both adherence and treatment completion [13]. For countries with a low TB incidence, current WHO’s guidelines recommend different options to choose from: daily INH for six months, daily RIF for 3–4 months, daily RIF plus INH for 3–4 months, and weekly RPT plus INH for three months [6]. 

The aim of the present study was to assess the completion and dropout rates of LTBI PT shifting regimen prescription from INH-alone to RIF-containing ones. 

## 2. Material and Methods

This study was carried out in the Regional TB Reference Centre/Villa Marelli Institute (VMI), Niguarda Ca’ Granda Hospital, Milan, Italy. We retrospectively selected individuals with a diagnosis of LTBI and the indication to start PT from 1st January 1992 to the 31st December 2018.

### 2.1. Latent Tuberculosis Infection Tests

During the first period of the study (from 1992 until December 2009), diagnosis of LTBI was based on the interpretation of a tuberculin skin test (TST), by using the Mantoux method (RT23 2 tuberculin units (TU) Serum Institut, Copenhagen, Denmark or Tubertest 5 TU, Sanofi Pasteur, Paris, France). According to the national guidelines [14,15], a test was considered to be positive in case of a local induration ≥5 millimetres (mm) for persons with the highest risk of developing active TB (i.e., persons with HIV infection or other immunosuppressive conditions, persons that had recent close contact with infectious TB patients, or subjects with radiological signs of previous untreated pulmonary TB), and ≥10 mm for other groups with an increased probability of progression to active TB, such as recent immigrants (i.e., those who migrated within the last 5 years) coming from high incidence countries, homeless individuals requiring admission to municipal shelters, people who inject drugs (PWID), and residents or employees of high-risk congregate settings (including healthcare workers (HCWs)). From December 2009, the interferon-γ release assay (IGRA) was adopted at VMI; in particular, the QuantiFERON^®^ Gold In-Tube (QIAGEN, Hilden, Germany) was employed until December 2015, when it was replaced by the QuantiFERON^®^ Gold Plus (LIAISON^®^- QuantiFERON^®^-TB Gold Plus, DiaSorin^®^, Saluggia, Italy).

### 2.2. Latent Tuberculosis Infection Diagnosis

A person with a LTBI diagnosis was classified as a recent TB contact or a not recent TB contact. Patients that did not have known contact with TB were also screened, according to national guidelines [14,15], if they belonged to the following groups:

- Homeless subjects who need a certificate to access municipal dormitories and shelters;
- Recently arrived asylum seekers/refugees;
- Irregular immigrants sent by non-governmental organizations (NGOs) operating in Milan;
- HCWs;
- Patients with autoimmune disorders that are starting immunosuppressive therapy (e.g., anti-tumour necrosis factor-α drugs);
- Adopted children from high TB incidence countries and their adoptive family;
- People working in the army and police;
- Housekeepers or workers in the food sector;
- Students arriving from high TB incidence countries or needing a certificate for abroad training;
- PWIDs sent by municipal services.

### 2.3. Preventive Treatment Regimens, Follow-Up and Outcomes

The first PT prescribed in the centre was INH-alone for six months [16]. Since 2007 shorter RIF-containing regimens (RIF alone for four months or in combination with INH for three months) have been prescribed, although they became part of routine prescriptions from 2009.

Before starting a PT, all patients underwent routine blood testing for cell count, liver and kidney function, glucose level, and HIV status. During follow-up, AEs were evaluated through patient’s self-reporting symptoms (e.g., headache, nausea) and blood tests, generally focused on assessing liver enzyme levels. Based on symptom severity, the patient’s general conditions, and lab abnormalities, a drug regimen could be momentarily stopped, definitively stopped, or changed [17].

Follow-up depended on the type of PT regimen (Table 1). However, individual schedules were adapted to pre-existing clinical conditions (e.g., elevated baseline serum level of transaminases) and/or to specific AEs. In case of a missed visit, patients were actively traced (e.g., by using phone calls) in order to arrange a new appointment in the following days.

The following treatment outcomes were adopted:

- Completion: uptake of at least 80% of the doses of one regimen, irrespectively of treatment changes, within 12 and 18 months from the start for RIF-containing and INH-alone, respectively [10];
- Treatment interruption: discontinuation prescribed by the attending physician (e.g., AEs, drug-susceptibility test result of index TB case showing drug-resistance, pregnancy, development of TB, or other unclear reasons);
- Loss to follow up: did not return for follow-up visits;
- Default: patient’s notification of his/her intention to interrupt PT;
- Death for any reason during PT;
- Unknown outcome: this outcome includes those who were transferred out.

Adverse events were grouped in the following six categories [17]:

- Mild-moderate liver disfunction: asymptomatic or symptomatic elevation of transaminase level (at least five or three folds, respectively);
- Severe hepatitis: symptomatic liver impairment requiring the patient’s hospitalization;
- Gastrointestinal system (GIS) disorders: abdominal pain, diarrhoea, and nausea;
- Central nervous system (CNS) disorders: headache, anxiety, insomnia, and depression;
- Peripheral neuropathy;
- Dermatological conditions: itching, rash, urticaria, angioedema, dermatitis, and eczema.

Patients were counselled to return to the centre in case they had symptoms suggestive for TB (coughing for more than two weeks, fever, night sweats, loss of weight) during the treatment, or any time after its completion.

### 2.4. Statistical Analysis

An ad hoc electronic form was created to collect all study variables. Qualitative data were summarized with absolute and relative frequencies. Medians and interquartile ranges were used to describe quantitative variables with a non-parametric, statistically proven distribution. Statistical comparison of non-normally distributed variables was performed using the Mann-Whitney test. Chi-squared or Fisher exact tests were performed to detect any statistical differences in the comparison of qualitative variables between INH- and RIF-containing regimens. A two-tailed p-value of less than 0.05 was considered statistically significant. The statistical software STATA version 16 (StataCorp, LLC, 4905 Lakeway Drive, Collage Station TX, USA) was used to perform statistical computations.

## 3. Results

A total of 20,734 records of individuals with LTBI diagnosis and the intention to start PT were present in the VMI database: 953 (4.6%) refused PT, whereas 110 (0.5%) were duplicated records and, therefore, excluded from the analysis. 

### 3.1. Reasons for Latent Tuberculosis Infection Assessment

LTBI assessment was performed because of recent TB contact (9333, 47.5%) or screening for other reasons identified by national guidelines (10,364, 52.7%) (Table 2). 

Screening programs were implemented for homeless persons admitted to municipal shelters (14.8%), irregular migrants sent by NGOs (8.3%), recently arrived asylum seekers/refugees (6.7%), HCWs (8.7%), and people with clinical risks for TB (7.1%).

Individuals receiving INH monotherapy included a higher proportion of irregular migrants (9.3%, *p* < 0.0001), HCWs (9.8%, *p* < 0.0001), patients with high TB risk (7.5%, *p* < 0.0001), and workers (6.6%, *p* < 0.0001). Individuals exposed to RIF-containing regimens included a higher proportion of homeless persons (30.4%, *p* < 0.0001) and asylum seekers/refugees (32.3%, *p* < 0.0001).

Before 2010 INH monotherapy was the main PT regimen (Table S1, Supplementary Material). Since then, RIF-based regimens were mainly prescribed in homeless persons (7.0% INH-alone vs. 31.2% RIF-based PT, *p* < 0.0001), irregular immigrants (4.2% INH-alone vs. 3.1% RIF-based PT, *p* = 0.004) and asylum seekers/refugees (7.0% INH-alone vs. 31.2% RIF-based PT, *p* < 0.0001) (Table S2, Supplementary Material).

### 3.2. Initial Treatment

**Figure 1:**
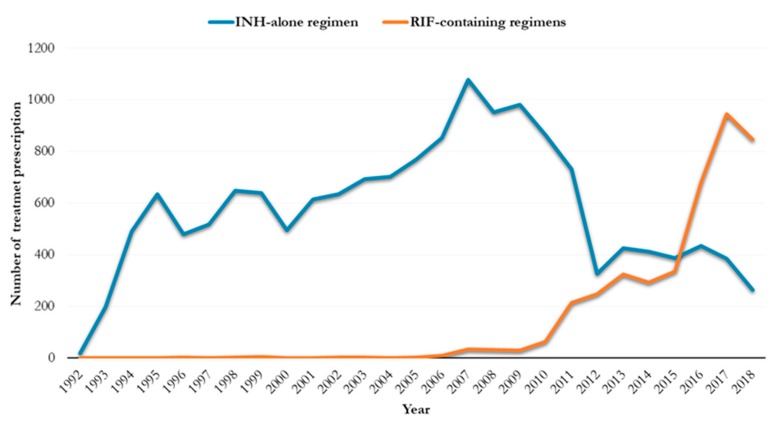
Change in LTBI treatment prescription from INH-alone to RIF-containing regimens (*n* = 19,670).

A total of 19,670 subjects with LTBI were initially treated either with INH monotherapy (15,605, 79.3%) or RIF-containing regimens (4065, 20.6%) (Figure 1), namely 531 (13.1%) RIF alone for four months and 3534 (86.9%) RIF plus INH for three months.

Individuals were mainly males (58.8%), foreign-born (73.3%), and with a median (IQR) age of 29 (23–37) years. Africa (30.0%), Southern America (19.5%), and Asia (14.7%) were the most represented geographical areas of origin (Table 3). In the group of individuals exposed to INH the proportion of males was lower (54.1%) than those under RIF-based PT (76.9%) (*p* < 0.0001), and the median (IQR) age was higher (30, 23–39) (*p* < 0.0001). Furthermore, the proportion of foreign-born subjects was lower in the INH group (69.6% vs. 87.3%, *p* < 0.0001). 

During the study period, changes in patients’ baseline characteristics have been found (Table S3, Supplementary Material). Since 2010, the male proportion (from 54.8% before 2010 to 64.4% after 2009, *p* < 0.0001) and the median age (from 29 years before 2010 to 30 years after 2009, *p* < 0.0001) increased. Furthermore, a rise in foreign-born patients (from 72.3% before 2010 to 74.6% after 2009, *p* < 0.0001), especially from Africa (from 25.2% before 2010 to 36.8% after 2009, *p* < 0.0001) have been reported.

Since 2010, the prescription of shorten RIF-based PT involved mainly young (median age of 36 years for INH-alone vs. 26 years for RIF-based PT, *p* < 0.0001), males (52.0% INH-alone vs. 77.6% RIF-based PT, *p* < 0.0001) and foreign-born (62.2% INH-alone vs. 88.0% RIF-based PT, *p* < 0.0001) (Table S4, Supplementary Material) patients. 

### 3.3. Treatment Outcomes

Treatment completion was high (79.4%), with differences between those starting with INH-alone (77.8%) and those with RIF-containing regimens (85.6%). The last had a 7.8% more likelihood of PT completion (*p* < 0.0001). 

Homeless persons showed the lowest completion rate (64.6%) (Table 4). Conversely, high rates were found for asylum seekers/refugees (91.0%) and for patients with a clinical risk (88.9%). An increased PT completion rate was described in the group treated with RIF-based regimen: homeless persons (76.8% vs. 55.6%, *p* < 0.0001), HCWs (93.3% vs. 77.8%, *p* < 0.0001), and recent TB contacts (89.8% vs. 81.7%, *p* < 0.0001).

Individuals who received INH-alone had a lower completion rate (77.9%) in comparison with those taking RIF-based regimens (85.7%) (*p* < 0.0001). In both PT groups loss to follow-up was high (71.4% INH-alone vs. 67.5% RIF-based, *p* = 0.06) (Table 5). Treatment interruption following a clinician decision was higher in those treated with INH-alone regimen (12.1% vs. 6.4%, *p* < 0.0001).

Adverse events were reported in 2631 patients, and were more frequent reported in those who received INH monotherapy (12.8% vs. 8.7%, *p* < 0.0001), mild-moderate liver impairment (5.5% vs. 1.3%, *p* < 0.0001) and peripheral neuropathy (0.8% vs. 0.2%, *p* < 0.0001). Dermatological disorders were most frequently found in those exposed to RIF-containing regimens (0.9%).

In general, in those who developed AEs during PT—regardless of the initially prescribed drug regimen—treatment completion increased when treatment changes were made (68.9% in who did not change PT vs. 81.1% in who changed PT, *p* < 0.0001) (Table S5, Supplementary Material). These treatment changes increased the possibility of a reduction in defaulters (11.2% in who did not change PT vs. 3.1% in who changed PT, *p* < 0.0001) and PT suspensions (16.1% in who did not change PT vs. 11.3% in who changed PT, *p* = 0.02).

Among those who changed the prescribed PT regimen (Table 6), no differences were observed either in treatment completion (72.5% from INH-alone to RIF-based vs. 74.0% from RIF-based regimens to INH-alone, *p* = 0.82) or in AEs development (77.7% from INH-alone to RIF-based vs. 86.0% from RIF-based regimen to INH-alone, *p* = 0.18).

Reasons for treatment discontinuation varied, with a higher rate of suspension reported in those switching from INH-alone to RIF-based PT (77.7% from INH-alone to RIF-based vs. 30.8% from RIF-based regimens to INH-alone, *p* = 0.001), while the rate of being lost in follow-up was higher in those who changed PT from an RIF-based to an INH-alone regimen (4.0% from INH-alone to RIF-based vs. 53.9% from RIF-based regimens to INH-alone, *p* < 0.001).

The development of asymptomatic transaminase elevation was higher in those who switched regimen from INH-alone to RIF-based PT (40.3% from INH-alone to RIF-based vs. 14.0% from RIF-based regimens to INH-alone, *p* < 0.001), whereas GIS problems were more frequent in those who changed from RIF-based PT to INH-alone (9.3% from INH-alone to RIF-based vs. 30.0% from RIF-based regimens to INH-alone, *p* < 0.001).

### 3.4. Dropout

**Figure 2:**
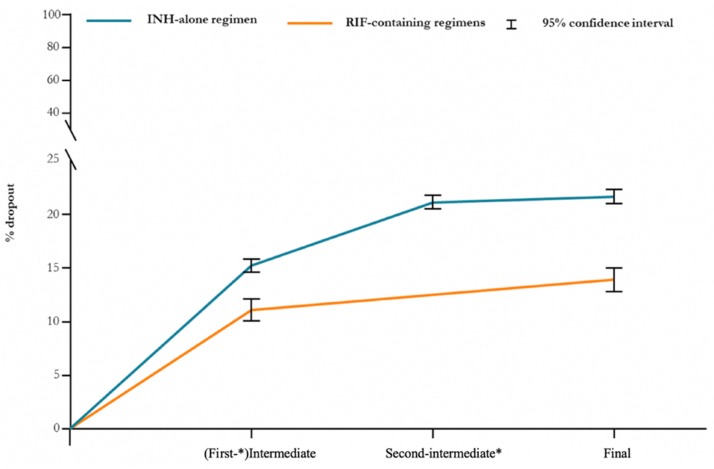
Dropout rate in individuals exposed to PT regimens (*n* = 19,159). * Refers to an INH-alone regimen.

The majority of patients discontinued PT in a time period relative to the first-intermediate follow-up visit (Figure 2).

Namely, 15.2% (95% confidence interval (CI), 14.6–15.8%) of those who received a INH-alone treatment dropped out compared to 11.1% (95% CI, 10.1–12.1%) under RIF-containing regimens. Dropout rates of INH monotherapy have decreased in recent years, since the introduction of RIF-based PT, from 22.8% before 2010 to 18.3% after 2009 (*p* < 0.0001) (Table S7, Supplementary Material).

Higher completion rates with RIF-based PT were also observed in some groups such as recent TB contacts (17.9% INH-alone vs. 9.7% RIF-based) (Table S6 and Figure S1, Supplementary Material), homeless persons (44.1% INH-alone vs. 22.9% RIF-based) (Table S6 and Figure S2, Supplementary Material), and HCWs (21.9% INH-alone vs. 6.1% RIF-based) (Table S6 and Figure S3, Supplementary Material). Notably, homeless persons displayed the highest dropout rates at the first-intermediate follow-up visit (2nd month for INH-alone and 1st month RIF-based regimens), reaching 31.7% (95% CI, 29.5–33.9%) for an INH-alone regimen vs. 20.6% (95% CI, 18.3–22.9%) for RIF-containing ones.

### 3.5. Tuberculosis Reactivation

Only six subjects which received INH-alone developed TB during PT.

A total of 293 patients (1.49%) who previously completed or interrupted PT returned complaining of TB symptoms: 60 (0.3%) were diagnosed with TB. In particular, TB occurred in 57 (0.4%) individuals who received INH-alone and in three individuals (0.1%) who had previously been treated with RIF-based PT; 37 (0.9%) discontinued PT.

## 4. Discussion

Globally, huge efforts have been made to tackle the TB epidemic following the adoption of the End TB Strategy by the international community. Unfortunately, some countries and the world as a whole are missing many of the targets set for the year 2020 [1]. Notably, the least implemented action so far is TB preventive treatment. Such a practice takes on even more importance for high-income countries where reactivation of LTBI is estimated to be responsible of most TB cases, especially affecting those who are marginalized [8,18].

A lack of organization of preventive programs due to inadequate political commitment, funding, and monitoring [19], as well as misperceptions of the TB risk in asymptomatic individuals and long PT duration are the main factors behind this clinical and public health lag [20]. Reducing factors that hinder further increase of PT completion rates is crucial to decrease the burden of disease.

We showed that RIF-containing regimens (mostly three months of daily RIF plus INH) are associated with a higher completion rate if compared with six months of daily INH (~10%). This difference was relevant in those suffering socio-economic hardship [17], such as homeless people, and in HCWs [21]. However, some confounders may have affected our results as patients with previously poor completion rates, such as homeless persons, have been given shortened regimen options since 2010. Furthermore, HCWs are known to be more compliant when LTBI diagnosis is proven by IGRA testing [21], which was introduced in clinical practice at VMI at the end of 2009.

In contrast to previous reports [22], AEs were lower in cases treated with RIF-containing—in which the INH plus RIF regimen was the most prescribed—rather than INH monotherapy. Severe hepatitis was negligible in both groups.

In our cohort, the majority of dropouts occurred at some unknown point in time between the initial visit and the (first-)intermediate appointment, that was at 30 days from start in RIF based regimens and at 60 days in the INH-alone group. The higher dropout rate at the first follow-up visit in those who received INH-alone could also be explained by the fact that the first appointment was more distant from the start than in the RIF-group. These data suggest that shorter PT regimens (e.g., one month of daily RPT plus INH [23]) could be helpful, but they might be not sufficient to address the issue of early drop-outs.

PT initiation and adherence could be strengthened by a dedicated and trained staff. Its goal should be to improve PT completion by providing patients with support and properly managing drug-induced side effects. Tools to enhance completions should include adherence coaching and cultural interventions [13,24], with the help of cultural mediator, especially for foreign-born persons living under conditions of social-economic hardship. On the other hand, a proper management of AEs development can be implemented by offering symptomatic treatments and/or by offering a different drug regimen in order to make PT more bearable.

Moreover, since the RIF-based PT—crowned by higher completion rates—implementation after 2009, the completion rates of the INH monotherapy increased (Table S7, Supplementary Materials). Many factors can explain these indirect achievements such as: (1) changes in the reasons for LTBI assessment (Tables S1 and S2, Supplementary Material) and patients’ demography (Table S3, Supplementary Material) during the study period, (2) an improvement on VMI staff in dealing with PT prescription and management during the years, and (3) the existence of shorten PT regimens (RIF-based)—after 2009—that may be more suitable for those suffering from social-economic hardship.

However, some limitations and confounders exist in our study. During the study period, the cohort changed its characteristics because of changes in policies, migration, and other temporal changes (Table S3, Supplementary Material). Screening for TB was compulsory for some categories of workers when the only available PT option was INH-alone. South American immigration to Milan occurred mostly between 1990 and 2005, whereas African immigrants (including asylum seekers/refugees) increased during the last decade, reaching a peak in 2016–2017 with massive inflows across Mediterranean Sea. Immigration from South America occurred when PT was only based on INH, while inflows from Africa happened after the implementation of shortened RIF-based regimens. Furthermore, as a consequence of the European resettlement scheme’s introduction for asylum seekers/refugees in 2015 (European Agenda on Migration of 13th May 2015), we preferred shortened schemes [8] in order to end PT before dislocation.

Nevertheless, in our study we found a high treatment completion rate among refugees/asylum seekers (91%), which is not in line with another Italian study [25], possibly because of organizational differences and a higher use of an RIF-containing shorter regimen in our study [8].

The number of patients who returned with TB symptoms after previous PT was very low, suggesting an appropriate assessment for signs and symptoms of TB disease and LTBI management. However, the high mobility of foreign-born persons within and outside national borders and the presence of numerous hospitals in Milan might have decreased our ability of detection.

In general, 15619 individuals completed their therapy. Thus, assuming a mean protective effect of 75% (range 60–90%) [3] and a 5–10% life-time risk of LTBI reactivation [26], the prescribed PT might have prevented 585–1170 new TB cases.

## 5. Conclusions

In light of our 27-years of clinical experience in providing TB preventive therapy, further shortening of PT regimens such the one based on a combination of RPT and INH is needed. This regimen would probably be suitable for enhancing completion in marginalised individuals, although cultural interventions and patient counselling to sustain PT adherence by trained staff should be implemented.

## Figures and Tables

**Table 1:** Schedule for clinic appointments to monitor latent TB infection (LTBI) treatment follow-up.

Selected Drug Regimen	Label	Appointment Number	Days from Starting Treatment
INH-alone	First-intermediate	1	60 days
	Second-intermediate	2	120 days
	Final	3	180 days
RIF-containing	Intermediate	1	30 days
	Final	2	90 days

INH, isoniazid; RIF, rifampicin.

**Table 2:** Reasons behind the assessment of LTBI treated in groups treated with INH-alone or RIF-containing regimens (*n* = 19,670).

	Total	Patients Initially Treated with INH-Alone Regimen	Patients Initially Treated with RIF-Containing Regimens	
	*n* (%)	*n* (%)	*n* (%)	*p*-Value ^&^
Total	19,670	15,605	4065	
LTBI assessment				
Recent TB contact ^†^	9333 (47.5)	8393 (53.8)	940 (23.1)	<0.0001
Screening programs for:				
Homeless	2908 (14.8)	1673 (10.7)	1235 (30.4)	<0.0001
Irregular migrants	1626 (8.3)	1455 (9.3)	171 (4.2)	<0.0001
Asylum seekers/refugees	1318 (6.7)	5 (0.03)	1313 (32.3)	<0.0001
HCWs	1701 (8.7)	1531 (9.8)	170 (4.2)	<0.0001
Clinical risk groups	1391 (7.1)	1162 (7.5)	229 (5.6)	<0.0001
School	338 (1.7)	331 (2.1)	7 (0.2)	<0.0001
Workers	1033 (5.3)	1032 (6.6)	1 (0.02)	<0.0001
Adoption	18 (0.1)	16 (0.1)	2 (0.1)	0.56
Military service	9 (0.1)	6 (0.04)	3 (0.1)	0.40
PWID	22 (0.1)	21 (0.1)	1 (0.02)	0.07

^†^ Any patients could be classified to have experienced recent tuberculosis contact or not. ^&^ Statistical comparisons between patients initially treated with INH-alone and with RIF-containing regimens in population groups. HCW, healthcare workers; INH, isoniazid; LTBI, latent tuberculosis infection; PWID, people who injects drugs; RIF, rifampicin; TB, tuberculosis.

**Table 3:** Baseline characteristics of patients with LTBI initially treated with INH-alone and RIF-containing regimens (*n* = 19,670).

	Total	Patients Initially Treated with INH-Alone Regimen	Patients Initially Treated with RIF-Containing Regimens	
	*n* (%)	*n* (%)	*n* (%)	*p*-Value
Total	19,670	15,605	4065	
Sex				
Males	11,562 (58.8)	8435 (54.1)	3127 (76.9)	<0.0001
Females	8108 (41.2)	7170 (46.0)	938 (23.1)	
Median (IQR) age ^†^	29 (23–37)	30 (23–39)	26 (20–33)	<0.0001
Nationality				
Foreign-born	14,414 (73.3)	10,864 (69.6)	3550 (87.3)	<0.0001
Geographical area of origin				
Italy	5256 (26.7)	4741 (30.4)	515 (12.7)	<0.0001
Western Europe/Northern America	19 (0.1)	13 (0.1)	6 (0.2)	0.24
Eastern Europe	1744 (8.9)	1465 (9.4)	279 (6.9)	<0.0001
Southern America	3843 (19.5)	3555 (22.8)	288 (7.1)	<0.0001
Asia	2900 (14.7)	2292 (14.7)	608 (15.0)	0.66
Africa	5907 (30.0)	3539 (22.7)	2368 (58.3)	<0.0001
Unknown	1 (0.0)	-	1 (0.0)	-

^†^ Data are not available for 91 (0.5%) patients. INH, isoniazid; IQR, interquartile range; LTBI, latent tuberculosis infection; RIF, rifampicin; TB, tuberculosis.

**Table 4:** Comparison of treatment completion rates between in individuals without treatment changes (*n* = 17,859).

	General Treatment Completion	Treatment Completion in Patients Treated with INH-Alone Regimen	Treatment Completion in Patients Treated with RIF-Containing Regimens	
	*n* (%)	*n* (%)	*n* (%)	*p*-Value
LTBI assessment groups				
Recent TB contact ^†^	7491/9080 (82.5)	6660/8155 (81.7)	831/925 (89.8)	<0.0001
Homeless persons	1867/2891 (64.6)	926/1665 (55.6)	941/1226 (76.8)	<0.0001
Irregular migrants	1181/1624 (72.7)	1060/1453 (73.0)	121/171 (70.8)	0.54
Asylum seekers/refugees	1193/1311 (91.0)	5/5 (100.0)	1188/1306 (91.0)	1.00
HCWs	1326/1671 (79.4)	1173/1507 (77.8)	153/164 (93.3)	<0.0001
Clinical risk	1139/1282 (88.9)	941/1067 (88.2)	198/215 (92.1)	0.10

^†^ Any patients could be classified to be recent tuberculosis contact or not. HCW, healthcare workers; INH, isoniazid; LTBI, latent tuberculosis infection; RIF, rifampicin; TB, tuberculosis.

**Table 5:** Comparison of treatment outcomes and AEs between RIF-containing regimens and INH-alone regimen without therapy changes (*n* = 19,253).

	Total	Patients Treated with INH-Alone Regimen	Patients Treated with RIF-Containing Regimens	
	*n* (%)	*n* (%)	*n* (%)	*p*-Value
	19,253	15,238	4015	
Treatment completion		11,877 (77.9)	3439 (85.7)	<0.0001
Reason for treatment discontinuation ^†^				
Lost to follow-up	2787 (70.8)	2398 (71.4)	389 (67.5)	0.06
Default	621 (15.8)	521 (15.5)	100 (17.4)	0.26
Suspension	445 (11.3)	408 (12.1)	37 (6.4)	<0.0001
Unknown	76 (1.9)	28 (0.8)	48 (8.3)	<0.0001
Died	8 (0.2)	6 (0.2)	2 (0.4)	0.41
Adverse events ^‡^	2303 (12.0)	1954 (12.8)	349 (8.7)	<0.0001
Transaminase elevation	884 (4.6)	833 (5.5)	51 (1.3)	<0.0001
Severe hepatitis	62 (0.3)	56 (0.4)	6 (0.2)	0.03
GIS problems	432 (2.2)	342 (2.2)	90 (2.2)	0.99
CNS problems	443 (2.3)	383 (2.5)	60 (1.5)	<0.0001
Peripheral neuropathy	134 (0.7)	125 (0.8)	9 (0.2)	<0.0001
Dermatological events	94 (0.5)	60 (0.4)	34 (0.9)	<0.0001

^†^ Percentages were calculated for those who did not completed preventive treatment. ^‡^ Each subject could report more than one adverse event. CNS, central nervous system; GIS, gastrointestinal system; INH, isoniazid; RIF, rifampicin.

**Table 6:** Comparison of treatment outcomes and AEs between groups treated with INH-alone and RIF-containing regimens who performed therapeutic changes (*n* = 417).

	INH-Alone Regimen Switch to RIF	RIF-Containing Regimens Switch to INH	
	*n* (%)	*n* (%)	*p*-Value
	367	50	
Treatment completion	266 (72.5)	37 (74.0)	0.82
Reason for treatment discontinuation ^†^			
Lost to follow-up	4 (4.0)	7 (53.9)	<0.0001
Default	11 (10.9)	0 (0.0)	0.36
Suspension	78 (77.2)	4 (30.8)	0.001
Died	0 (0.0)	0 (0.0)	-
Unknown	8 (7.9)	2 (15.4)	0.32
Adverse events ^‡^	285 (77.7)	43 (86.0)	0.18
GIS problems	34 (9.3)	15 (30.0)	<0.0001
CNS problems	44 (12.0)	6 (12.0)	1.00
Transaminase elevation	148 (40.3)	7 (14.0)	<0.0001
Dermatological events	22 (6.0)	6 (12.0)	0.11
Peripheral neuropathy	13 (3.5)	1 (2.0)	1.00
Severe hepatitis	21 (5.7)	0 (0.0)	0.16

^†^ Percentages were calculated for those who did not completed preventive treatment. ^‡^ Each subject could report more than one adverse event. CNS, central nervous system; GIS, gastrointestinal system; INH, isoniazid; RIF, rifampicin.

## Supplementary Materials

The following are available online at https://www.mdpi.com/2077-0383/9/1/101/s1, Table S1. Reasons behind the assessment of LTBI treated in groups treated with INH-alone or RIF-containing regimens before 2010 (*n* = 11,490). Table S2. Reasons behind the assessment of LTBI treated in groups treated with INH-alone or RIF-containing regimens after 2009 (*n* = 8180). Table S3. Comparison of demographic characteristics between patient treated before 2010 and after 2009 (*n* = 19,670). Table S4. Comparison of demographic characteristics between patients treated with INH-alone and those with RIF-containing regimens after 2009 (*n* = 8180). Table S5. Comparison of treatment outcomes in patients with adverse events without and with therapy changes (*n* = 2631). Table S6. Dropout rates in individuals exposed to preventive treatment, without changes of drug regimen. Table S7. Dropout rates in individuals exposed to preventive treatment with INH-alone regimen before 2010 and after 2009. Figure S1. Dropout rate in recent TB contacts (*n* = 9076). Figure S2. Dropout rate in homeless (*n* = 2879). Figure S3. Dropout rate in health care workers (*n* = 1664).

## Abbreviations

AE	adverse event
CNS	central nervous system
GIS	gastrointestinal system
HCW	health care worker
HIV	human immunodeficiency virus
IGRA	interferon-γ release assay
INH	isoniazid
IQR	interquartile range
LTBI	latent tuberculosis infection
NGO	non-governmental organization
PT	preventive treatment
PWID	people who inject drugs
RIF	rifampicin
RPT	rifapentine
TB	tuberculosis
TST	tuberculin skin test
TU	tuberculin units
VMI	Villa Marelli Institute
WHO	World Health Organization
