# Supplementary material for: Latent Tuberculosis Infection Treatment Completion while Shifting Prescription from Isoniazid-Only to Rifampicin-Containing Regimens: A Two-Decade Experience in Milan, Italy
Source: J Clin Med. 2019 Dec 31;9(1):101. doi: 10.3390/jcm9010101 (PMC7019895; doi:10.3390/jcm9010101)
Supplement: Supplementary file 1 [file jcm-09-00101-s001.pdf]

Table S1. Reasons behind the assessment of LTBI treated in groups treated with INH-alone or RIF-containing regimens before 2010 (*n*=11490).

	Total	Patients initially treated with INH-alone regimen	Patients initially treated with RIF-containing regimens	
	n (%)	*n* (%)	*n* (%)	*p-value*[&]
**Total**	11490	11 373	117	
**LTBI assessment**				
Recent TB contact†	5920 (51.5)	5828 (51.2)	92 (78.6)	<0.0001
Screening programs for:				
Homeless	1378 (12.0)	1375 (12.1)	3 (2.6)	<0.0001
Irregular migrants	1330 (11.6)	1326 (11.7)	4 (3.4)	0.003
Asylum seekers/refugees	0 (0.0)	0 (0.0)	0 (0.0)	-
HCWs	1091 (9.5)	1086 (9.6)	5 (4.3)	0.05
Clinical risk groups	401 (0.5)	389 (3.4)	12 (10.3)	<0.0001
School	329 (2.9)	329 (2.9)	0 (0.0)	0.08
Workers	1030 (9.0)	1030 (9.1)	0 (0.0)	<0.0001
Adoption	7 (0.1)	7 (0.1)	0 (0.0)	1.00
Military service	0 (0.0)	0 (0.0)	0 (0.0)	-
PWID	20 (0.2)	20 (0.2)	1 (0.9)	0.19

† Any patients could be classified to be recent tuberculosis contact or not. [&]Statistical comparison between patients initially treated with INH-alone and with RIF-containing regimens. HCW, healthcare workers; INH, isoniazid; LTBI, latent tuberculosis infection; PWID, people who injects drugs; RIF, rifampicin; TB, tuberculosis.

Table S2. Reasons behind the assessment of LTBI treated in groups treated with INH-alone or RIF-containing regimens after 2009 (*n*=8180).

	Total	Patients initially treated with INH-alone regimen	Patients initially treated with RIF-containing regimens	
	n (%)	*n* (%)	*n* (%)	*p-value*[&]
**Total**	8180	4232	3948	
**LTBI assessment**				
Recent TB contact†	3413 (41.7)	2565 (60.6)	848 (21.5)	<0.0001
Screening programs for:				
Homeless	1530 (18.7)	298 (7.0)	1232 (31.2)	<0.0001
Irregular migrants	296 (3.6)	129 (3.1)	167 (4.2)	0.004
Asylum seekers/refugees	1318 (16.1)	5 (0.1)	1313 (33.3)	<0.0001
HCWs	610 (7.5)	445 (10.5)	165 (4.2)	<0.0001
Clinical risk groups	990 (12.1)	773 (18.3)	217 (5.5)	<0.0001
School	9 (0.1)	2 (0.1)	7 (0.2)	0.10
Workers	3 (0.04)	2 (0.05)	1 (0.03)	1.00
Adoption	11 (0.1)	9 (0.2)	2 (0.05)	0.07
Military service	9 (0.1)	6 (0.1)	3 (0.1)	0.51
PWID	1 (0.01)	1 (0.02)	0 (0.0)	1.00

† Any patients could be classified to be recent tuberculosis contact or not. [&]Statistical comparison between patients initially treated with INH-alone and with RIF-containing regimens. HCW, healthcare workers; INH, isoniazid; LTBI, latent tuberculosis infection; PWID, people who injects drugs; RIF, rifampicin; TB, tuberculosis.

Table S3. Comparison of demographic characteristics between patient treated before 2010 and after 2009 (*n* = 19670).

	Total	Patients treated before 2010	Patients treated after 2009	
	n (%)	*n* (%)	*n* (%)	*p-value*
**Total**	19670	11490	8180	
**Sex**				
Males	11562 (58.8)	6297 (54.8)	5265 (64.4)	<0.0001
Females	8108 (41.2)	5193 (45.2)	2915 (35.6)	
**Median (IQR) age**	29 (23–37)†	29 (23–35)‡	30 (22–43)^	<0.0001
**Nationality**				

Foreign-born	14414 (73.3)	8309 (72.3)	6105 (74.6)	<0.0001
**Geographical area of origin**				
Italy	5256 (26.7)	3181 (27.7)	2075 (25.4)	<0.0001
Western Europe /Northern America	19 (0.1)	1 (0.01)	18 (0.22)	<0.0001
Eastern Europe	1744 (8.9)	985 (8.6)	759 (9.3)	0.09
Southern America	3843 (19.5)	2727 (23.7)	1116 (13.6)	<0.0001
Asia	2900 (14.7)	1699 (14.8)	1201 (14.7)	0.84
Africa	5907 (30.0)	2897 (25.2)	3010 (36.8)	<0.0001
Unknown	1 (0.00)	-	1 (0.01)	-

†Data are not available for 91 (0.5 %) patients. ‡ Data are not available for 84 (0.7 %) patients. ^Data are not available for 7 (0.09 %) patients. INH, isoniazid; IQR, interquartile range; LTBI, latent tuberculosis infection; RIF, rifampicin; TB, tuberculosis.

Table S4. Comparison of demographic characteristics between patients treated with INH-alone and those with RIF-containing regimens after 2009 (*n*=8180).

	**Total**	**Patients initially treated with INH-alone regimen**	**Patients initially treated with RIF-containing regimens**	
	***n* (%)**	***n* (%)**	***n* (%)**	***p-value***
**Total**	8180	4232	3948	
**Sex**				
Males	5265 (64.4)	2200 (52.0)	3065 (77.6)	<0.0001
Females	2915 (35.6)	2032 (48.0)	883 (22.4)	
**Median (IQR) age†**	30 (22-43)	36 (25–51)	26 (20–32)	<0.0001
**Nationality**				
Foreign-born	6105 (74.6)	2630 (62.2)	3475 (88.0)	<0.0001
**Geographical area of origin**				
Italy	2075 (25.4)	1602 (37.9)	473 (12.0)	<0.0001
Western Europe /Northern America	18 (0.2)	12 (0.3)	6 (0.2)	0.21
Eastern Europe	759 (9.3)	487 (11.5)	272 (6.9)	<0.0001
Southern America	1116 (13.6)	861 (20.3)	255 (6.5)	<0.0001
Asia	1201 (14.7)	609 (14.4)	592 (15.0	0.44
Africa	3010 (36.8)	661 (15.6)	2349 (59.5)	<0.0001
Unknown	1 (0.01)	-	1 (0.03)	-

†Data are not available for 7 (0.09 %) patients. INH, isoniazid; IQR, interquartile range; LTBI, latent tuberculosis infection; RIF, rifampicin; TB, tuberculosis.

Table S5. Comparison of treatment outcomes in patients with adverse events without and with therapy changes (*n*=2631).

	**Patients with adverse events**	**Without therapy changes**	**With therapy changes**	
	***n* (%)**	***n* (%)**	***n* (%)**	**p-value**
	2631	2303	328	
Treatment completion	1852 (70.4)	1586 (68.9)	266 (81.1)	<0.0001
Lost to follow-up	90 (3.4)	84 (3.7)	6 (1.8)	0.09
Default	267 (10.2)	257 (11.2)	10 (3.1)	<0.0001
Suspension	408 (15.5)	371 (16.1)	37 (11.3)	0.02
Transfer out	2 (0.1)	2 (0.1)	0 (0.0)	1.00
Died	1 (0.04)	1 (0.04)	0 (0.00)	1.00
Unknown	11 (0.4)	2 (0.1)	9 (2.7)	<0.0001

Table S6. Dropout rates in individuals exposed to preventive treatment, without changes of drug regimen.

**Follow-up visits**	**INH-alone regimen *n*/N (%)**	**95% CI**	**RIF-containing regimens *n*/N (%)**	**95% CI**
**Total**				
(First-*)Intermediate	2297/15161	14.6–15.8	444/3997	10.1–12.1

	(15.2)		(11.1)	
Second-Intermediate*	3197/15161 (21.1)	20.5–21.8	–	–
Final	3280/15161 (21.6)	21.0–22.3	557/3997 (13.9)	12.8–15.0
**Homeless**				
(First-*)Intermediate	526/1658 (31.7)	29.5–33.9	252/1221 (20.6)	18.3–22.9
Second-Intermediate*	717/1658 (43.2)	40.8–45.6	–	–
Final	731/1658 (44.1)	41.7–46.5	280/1221 (22.9)	20.5–25.3
**HCWs**				
(First-*)Intermediate	246/1501 (16.4)	14.5–18.3	8/163 (4.9)	1.6–8.2
Second-Intermediate*	320/1501 (21.3)	19.2–23.4	–	–
Final	328/1501 (21.9)	19.8–24.0	10/163 (6.1)	2.4–9.8
**Recent TB contacts**				
(First-*)Intermediate	991/8155 (12.2)	11.5–12.9	41/921 (4.5)	3.2–5.8
Second-Intermediate*	1413/8155 (17.4)	16.6–18.2	–	–
Final	1454/8155 (17.9)	17.1–18.7	89/921 (9.7)	7.8–11.6

* Refers only to INH-alone regimen. CI, confident interval; HCW, health care worker; INH, isoniazid; RIF, rifampicin; TB, tuberculosis

Table S7. Dropout rates in individuals exposed to preventive treatment with INH-alone regimen before 2010 and after 2009.

Follow-up visits	INH-alone regimen *n*/N (%)	Patients treated before 2010 *n*/N (%)	Patients treated after 2009 *n*/N (%)	*p-value*
**Total**				
(First-)Intermediate	2297/15161 (15.2)	1812/11300 (16.0)	485/3861 (12.6)	<0.0001
Second-Intermediate	3197/15161 (21.1)	2507/11300 (22.2)	690/3861 (17.9)	<0.0001
Final	3280/15161 (21.6)	2574/11300 (22.8)	706/3861 (18.3)	<0.0001

INH, isoniazid

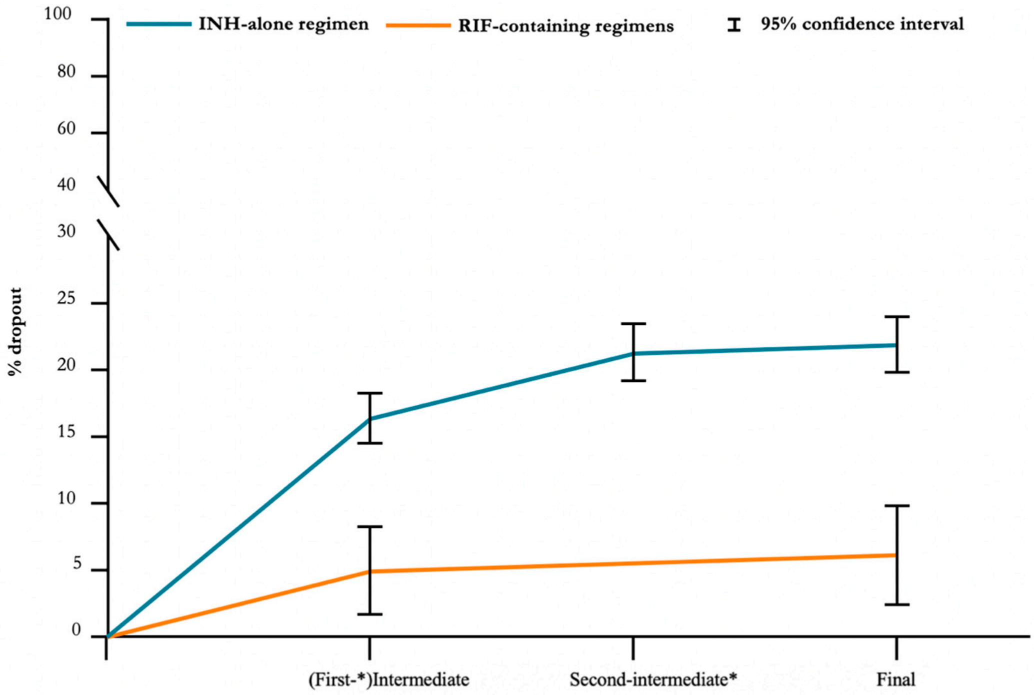

Figure S1. Dropout rate in recent TB contacts (*n*=9076). * Refers to INH-alone regimen.

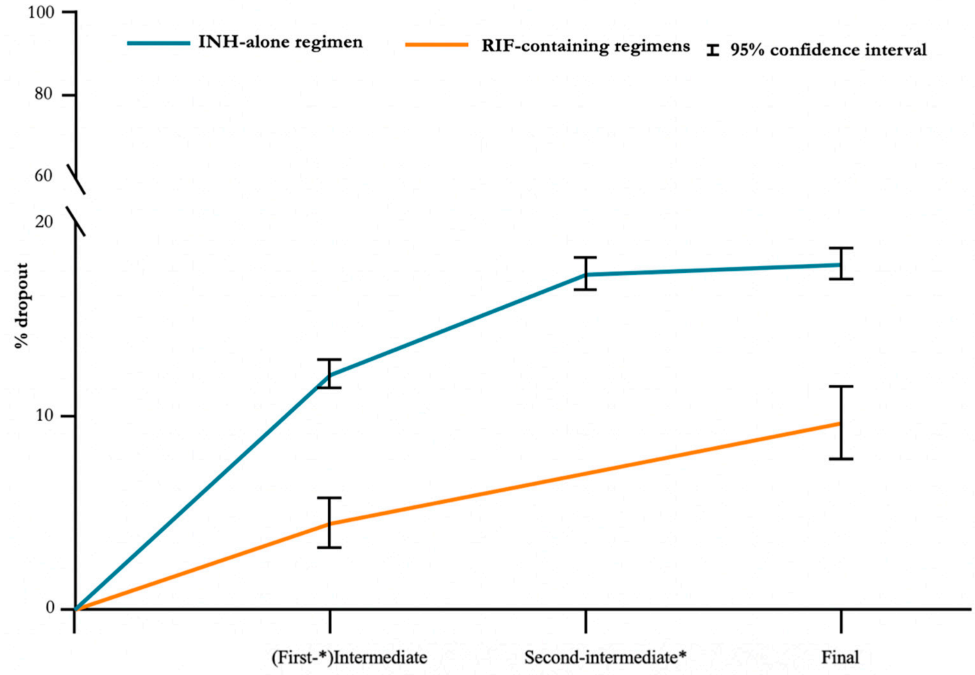

Figure S2. Dropout rate in homeless (n=2879). * Refers to INH-alone regimen.

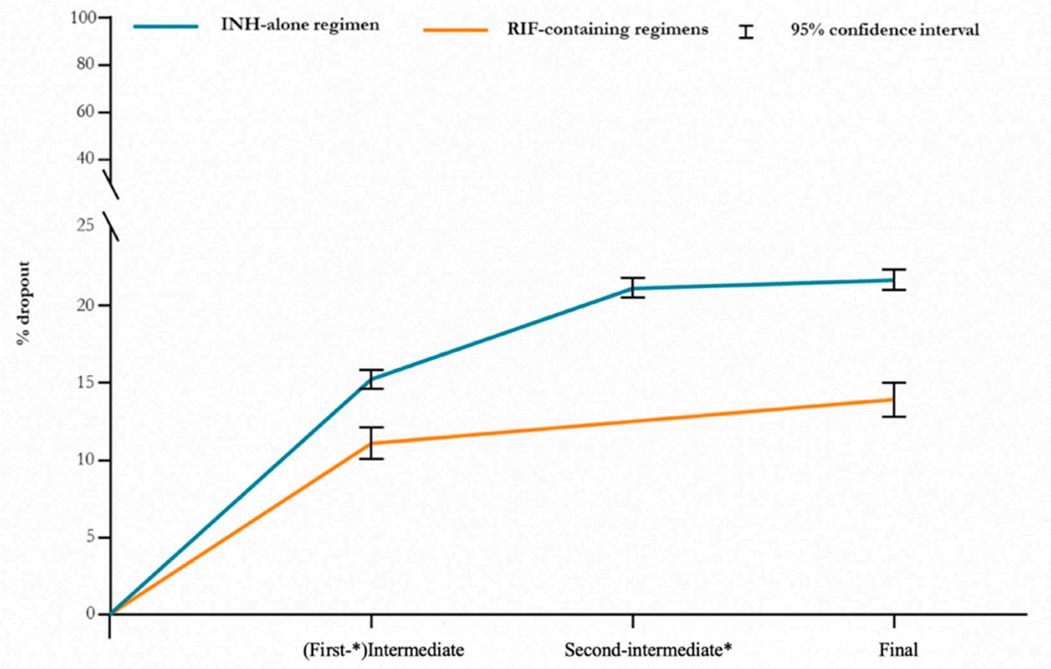

Figure S3. Dropout rate in health care workers (n = 1664). * Refers to INH-alone regimen.
